# Supplementary material for: A time transect of exomes from a Native American population before and after European contact
Source: Nat Commun. 2016 Nov 15;7:13175. doi: 10.1038/ncomms13175 (PMC5116069; doi:10.1038/ncomms13175)
Supplement: Supplementary Information — Supplementary Figures 1-12, Supplementary Tables 1-10, Supplementary Notes 1-8 and Supplementary References [file ncomms13175-s1.pdf]

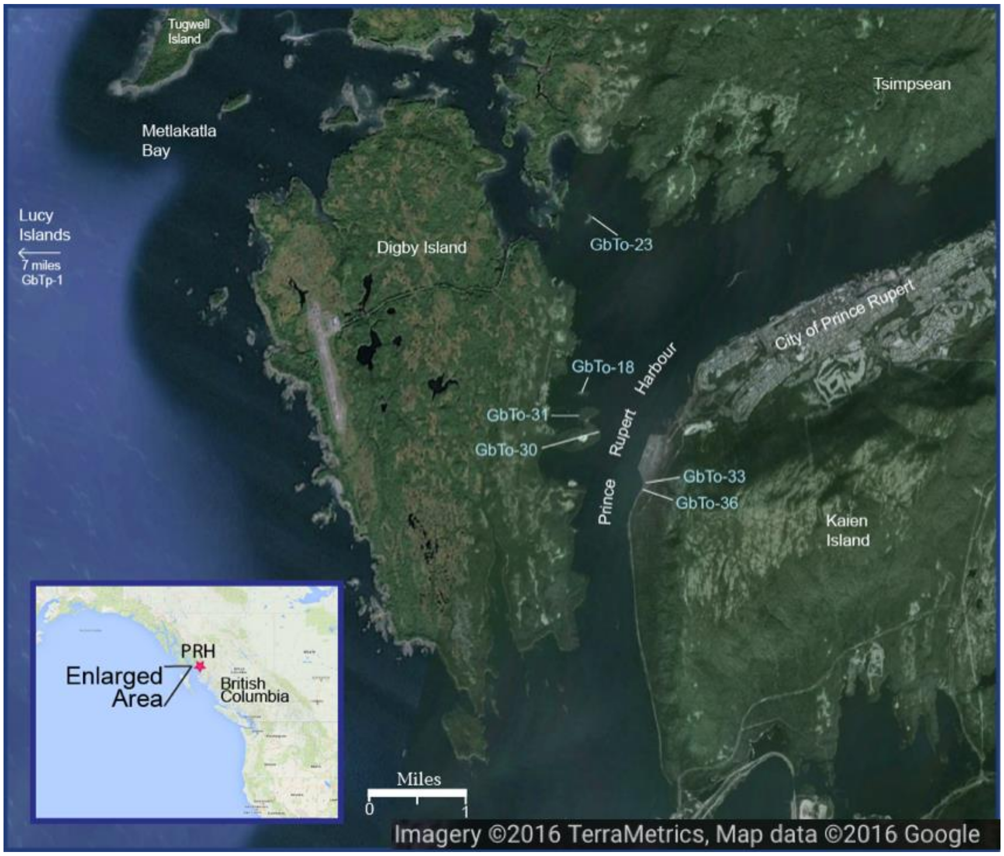

Supplementary Figure 1 | Archaeological sites in the Prince Rupert Harbour region of British Columbia, Canada.

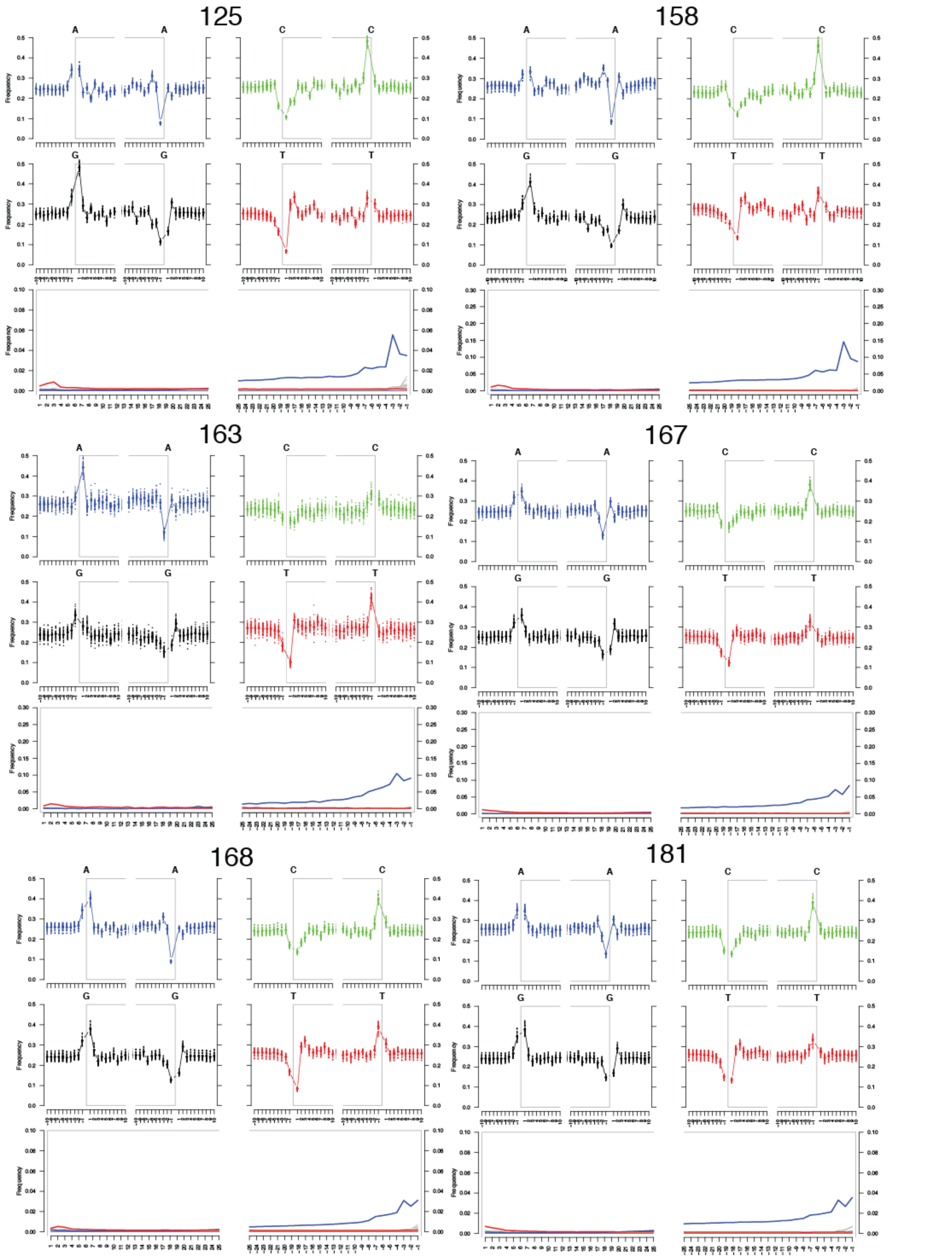

Supplementary Figure 2

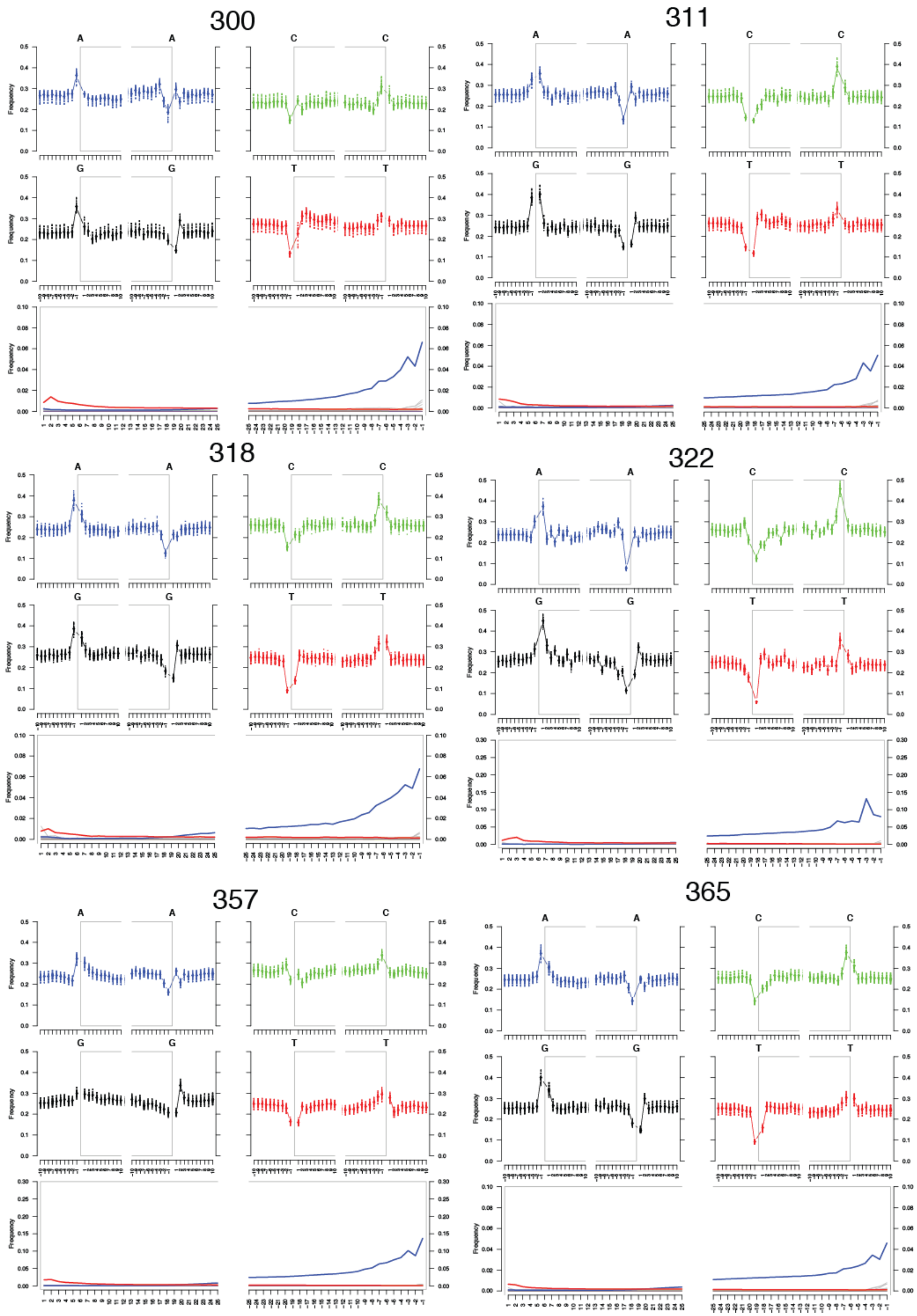

Supplementary Figure 2 (Continued)

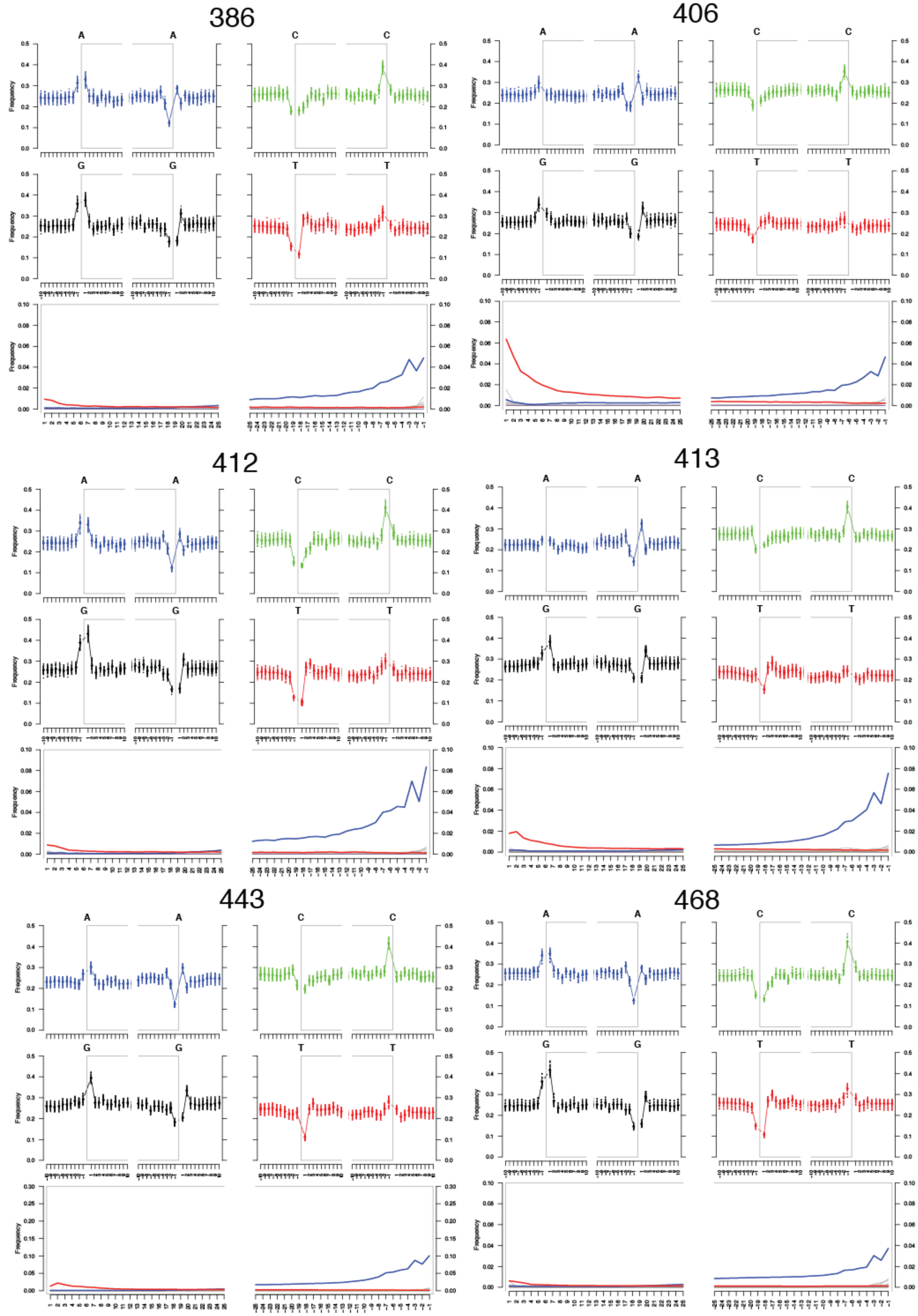

Supplementary Figure 2 (Continued)

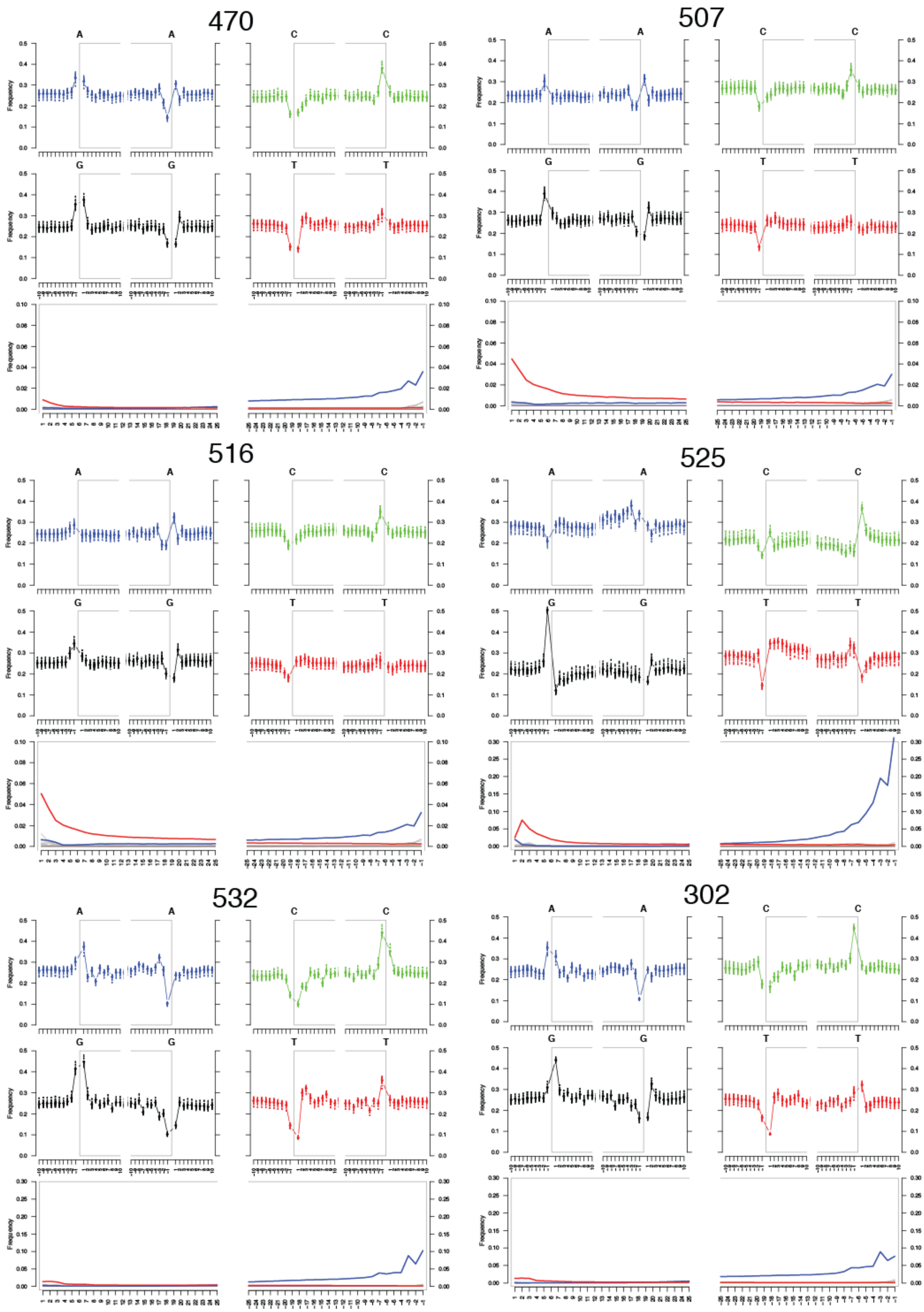

Supplementary Figure 2 (Continued)

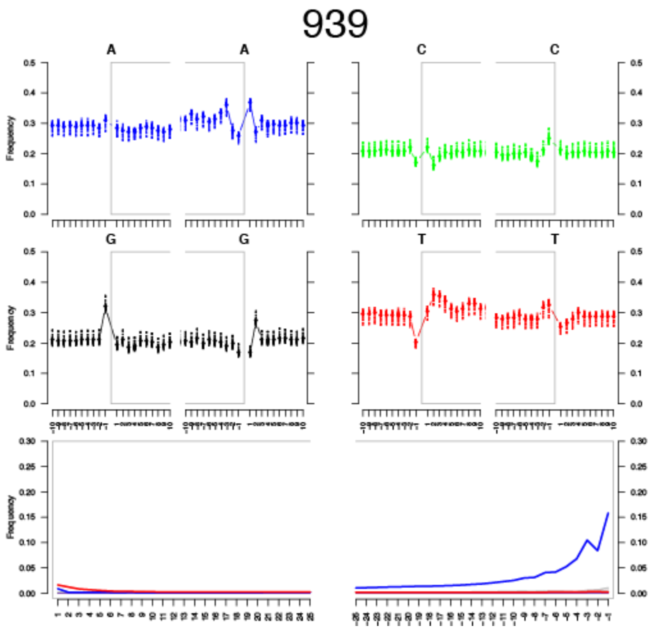

Supplementary Figure 2 | DNA damage patterns for the PRH Ancients. Random subset of all mapped reads for each PRH Ancient. The mismatch frequency is relative to the reference as function of read position, C→T in red and G→A in blue.

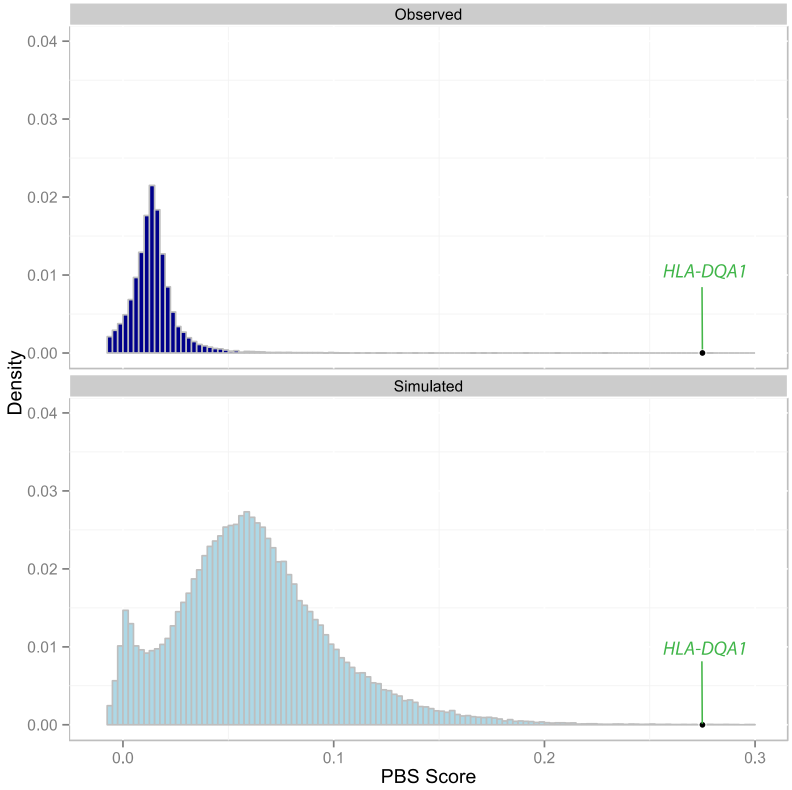

Supplementary Figure 3 | Distribution of PRH Ancient PBS scores simulated under neutrality and observed.

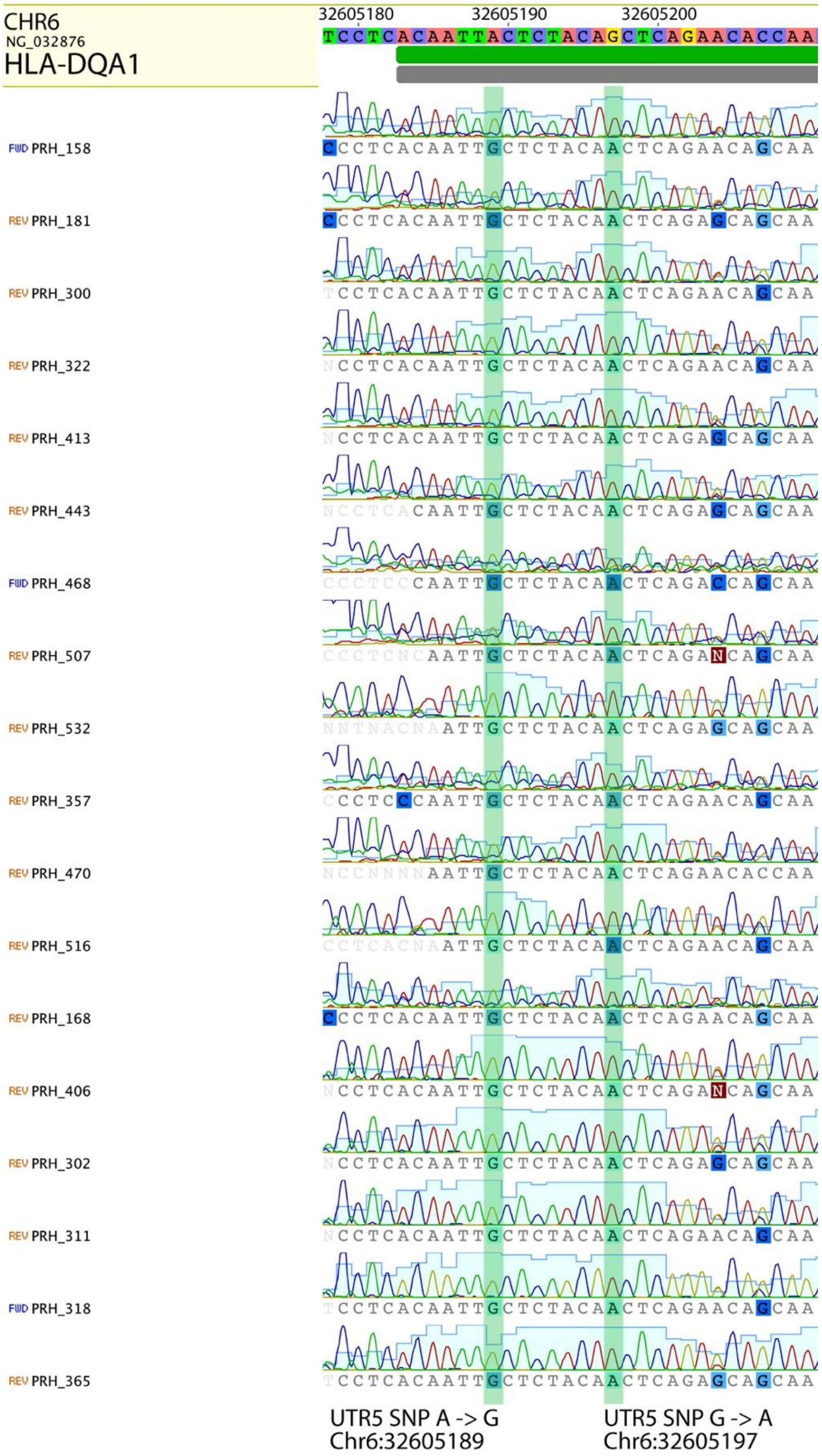

Supplementary Figure 4 | Confirmation of *HLA-DQA1* high frequency SNPs via Sanger sequencing in 18 of the PRH Ancients. Primers used for this region did not align to any other segment of the human genome via the UCSC In-Silico PCR.

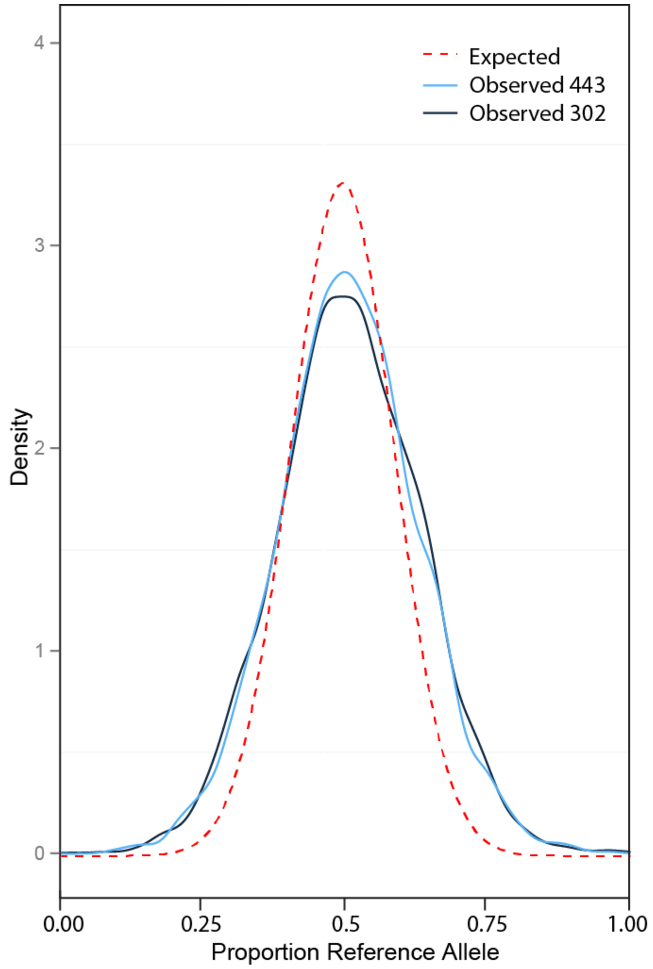

Supplementary Figure 5 | Assessment of capture reference bias. The solid line represents called heterozygous SNPs with more than 20 reads in the 2 ancient samples with the best coverage. The dashed line represents the expected distribution without bias. The two ancient samples show a slight bias towards the reference (both showing a mean proportion of ~0.56). This bias could also be attributed to differential mapping. However, this bias does not correlate with our top PBS hit, since the SNPs are all called for the *alternative* allele to the reference.

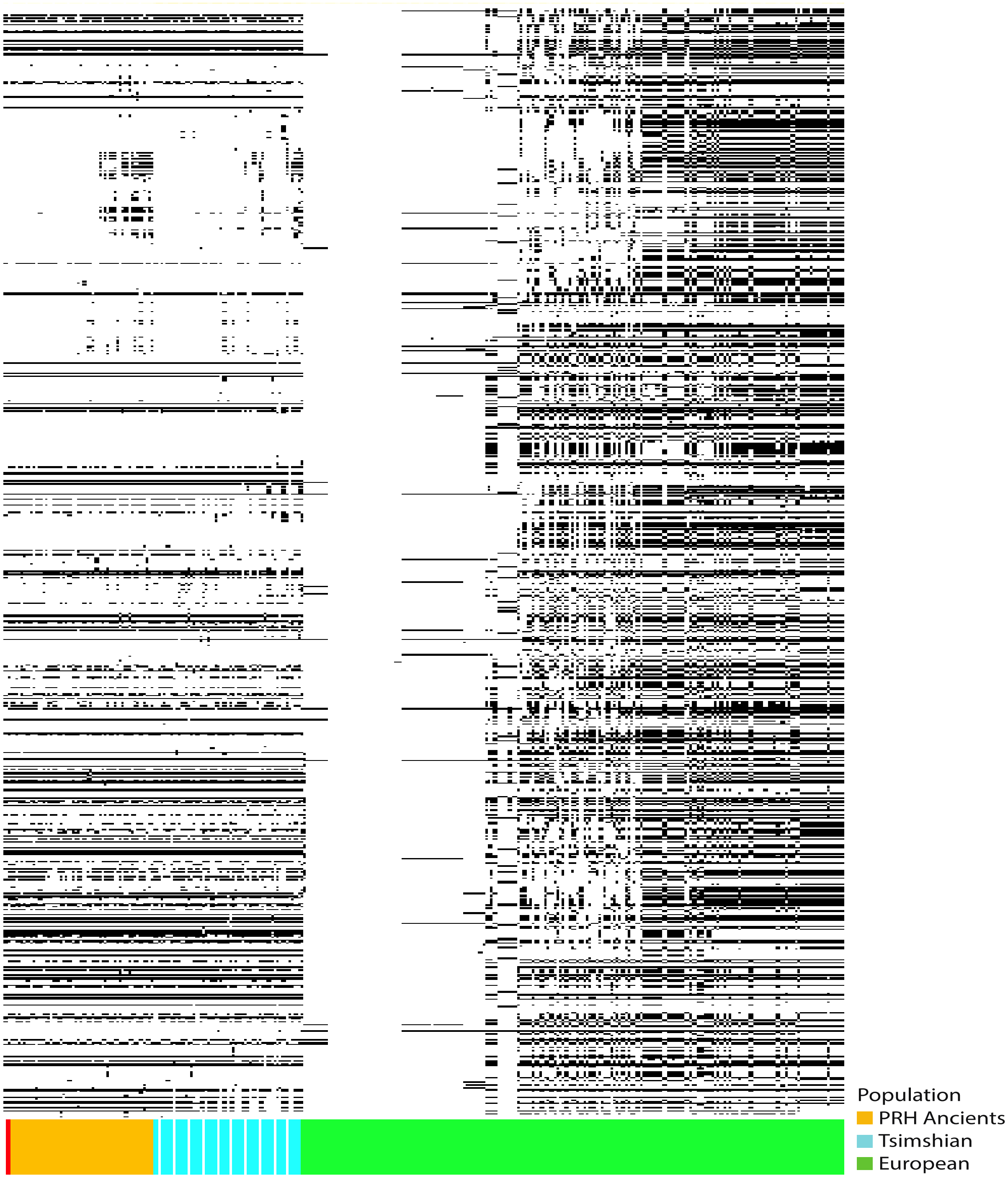

Supplementary Figure 6 | Haplotype visualization of *HLA-DQA1.* Haplotype graph of the *HLA-DQA1* gene. We counted the differences between a randomly chosen haplotype from ancient individual PRH 125 and all other haplotypes of PRH Ancients, modern Tsimshian, and the British (GBR) samples from the 1,000 Genomes Phase 3 data[1]. Each column is a haplotype, and the two haplotypes of individual 125 are the first two columns (indicated in red). Each row is a SNP. Black indicates a derived allele (allele that differs from the chimpanzee reference), and white indicates an ancestral allele (allele that is identical to the chimpanzee reference). Note that while this region has been portrayed to be a continuous segment, it is not, and we have simply concatenated the exons in this gene. The colors refer to the different populations considered in this plot, as indicated by the legend.

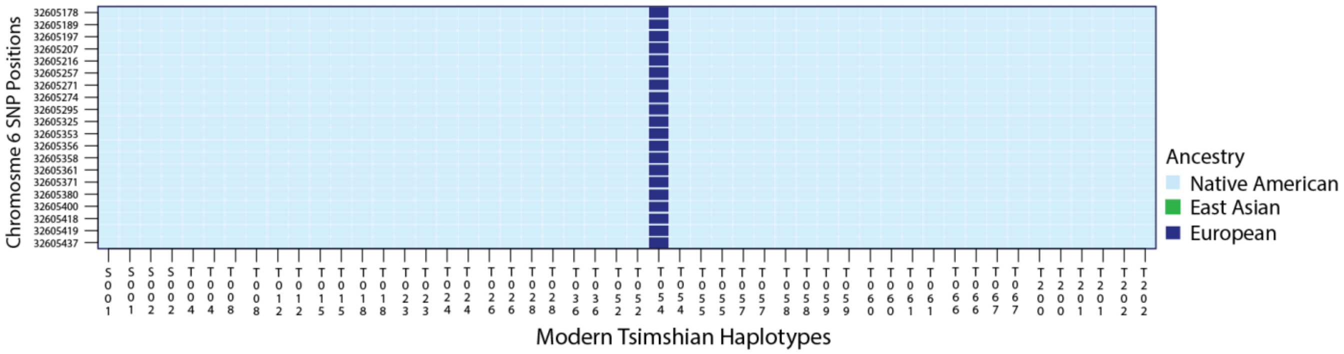

Supplementary Figure 7 | Local ancestry of *HLA-DQA1.* Ancestry components of the *HLA-DQA1* UTR5 region in the modern Tsimshian. We used RFMix[2] to examine the local ancestry across chromosome 6 in the modern Tsimshian, with the above figure highlighting the region where the highest SNP frequency changes are observed, with respect to the ancient population. Only one of the 50 haplotypes is predicted to be of European ancestry, suggesting that admixture cannot explain the frequency differences between the two populations.

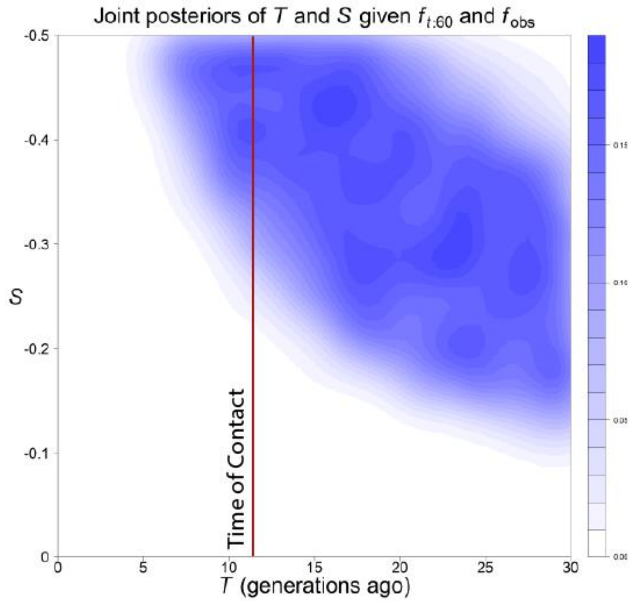

Supplementary Figure 8 | Joint posterior distribution of selection coefficients and time for negative selection model. Given the negative selection simulation results, we assumed that the best fit for our data is a model that includes a shift from positive selection in the PRH Ancients to negative selection in the modern Tsimshian, after European contact. We examined the values of time and strength of negative selection consistent with the change in allele frequencies observed between the modern and ancient individuals in *HLA-DQA1*. Time of contact is estimated to be 300 years ago (12 generations ago) for the Pacific Northwest.

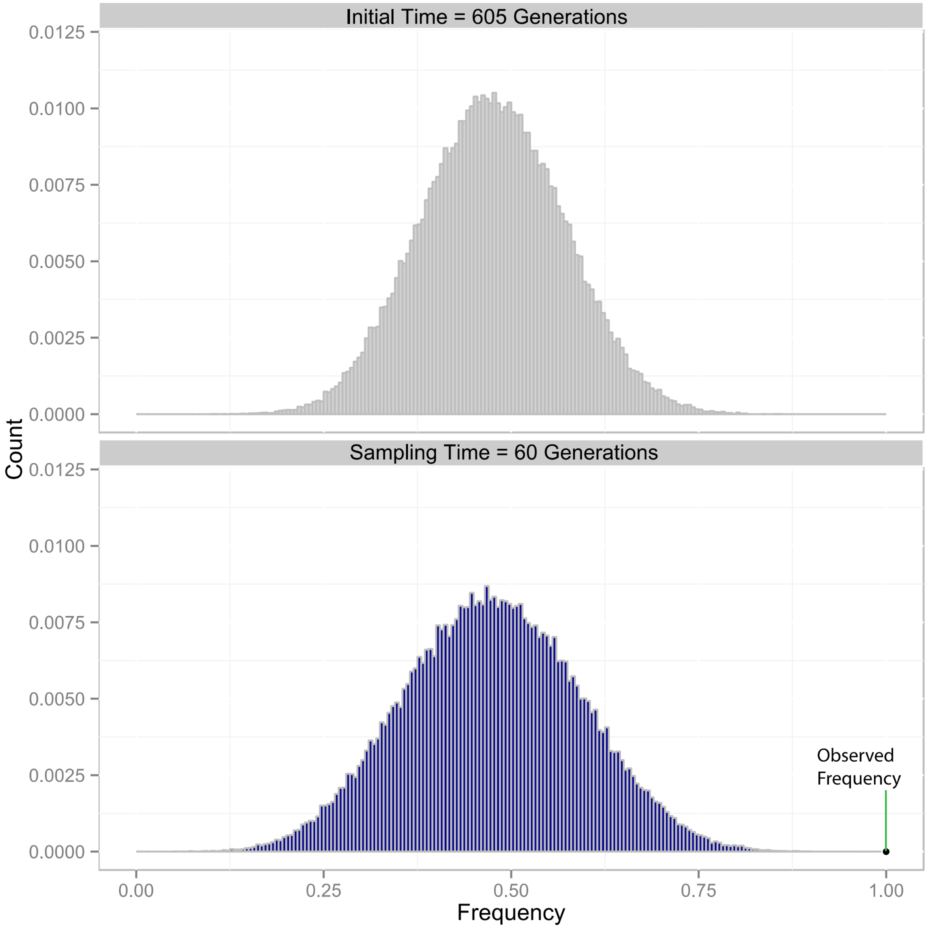

Supplementary Figure 9 | Simulations of *HLA-DQA1* allele frequencies under a neutral model. The light grey depicts the distribution of initial allele frequencies of the Native American population at the time of divergence from East Asian populations, using a backwards simulation conditioned on the present-day CHB frequency. The blue distribution depicts the allele frequencies at the sampling point of the ancient population. The neutral model is not a good a fit for our data, given the observed frequencies in the ancient population, which are nearly fixed in the UTR5 region of the *HLA-DQA1* gene.

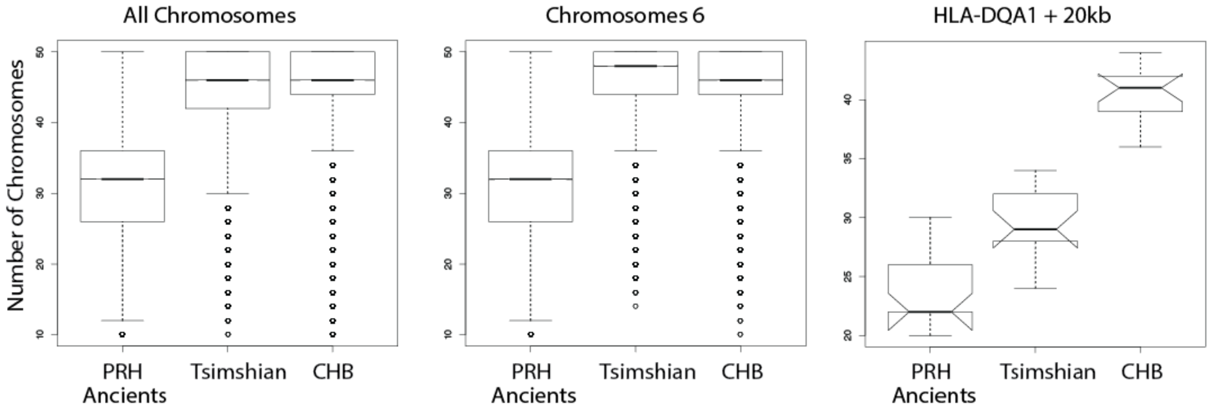

Supplementary Figure 10 | Comparison of missing data across populations. As expected from the degraded nature of ancient DNA, the PRH ancients exhibit more missing data than both modern populations. The level of missing data across the genome and across chromosome 6 is similar. The *HLA-DQA1* region shows that the coverage for PRH Ancients is less than that for the modern Tsimshian, and that the modern Tsimshian have less coverage than CHB. Though we observe a decrease in coverage in the modern Tsimshian and PRH ancients relative to the background level of coverage, the number of observed alleles is always greater than 20 (i.e., 10 diploid individuals), which is sufficient to compute accurate values of F_{ST} and is over twice as large as the minimal threshold of non-missing individuals (five diploids or 10 alleles) for calling an allele frequency in our *ANGSD* pipeline. Further, the high frequency variant identified using our PBS scan was confirmed by Sanger sequencing in 18 diploid individuals (36 total alleles) from the PRH Ancients (Supplementary Fig. 11), indicating that it is not sample size that is driving the observed PBS patterns.

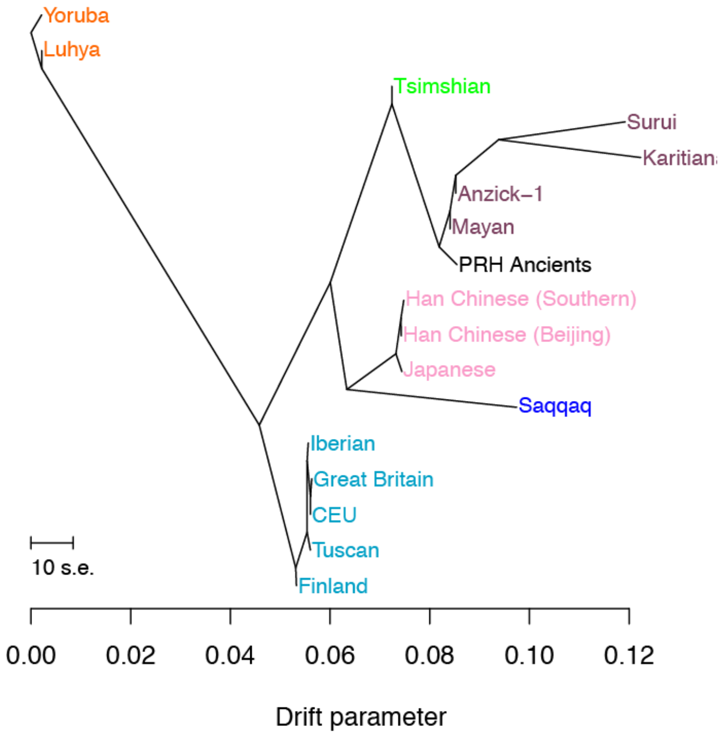

Supplementary Figure 11 | *TreeMix* with zero migration events. The modern Tsimshian population falls ancestral to the Native Americans and the PRH Ancients. This placement is due to European admixture into the modern Tsimshian. The modern Tsimshian form a sister group to the PRH Ancients when accounting for European admixture (Fig. 1c)

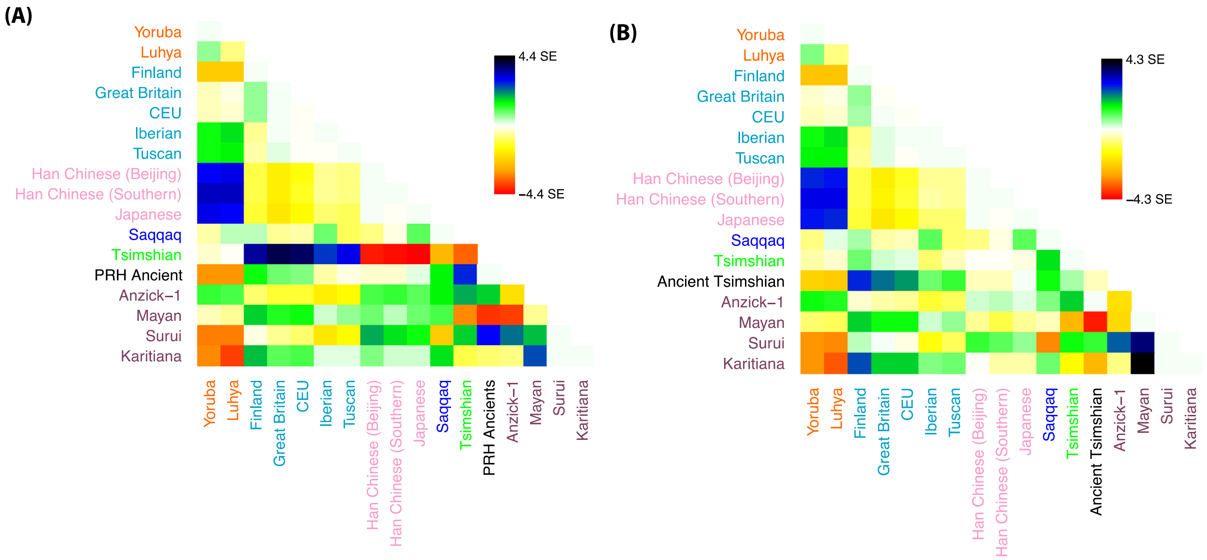

Supplementary Figure 12 | *TreeMix* residuals. (A) No migration events. (B) One migration event, leading to a reduction in the large residuals observed in the modern Tsimshian.

Supplementary Table 1 | PRH Ancient sample characterization

Catalog ID	Archaeo-logical site	Osteology-based sex estimation	Sequence-based sex estimation	Tested Specimen	Conv ^{14}C years BP	Cal years BP	Dated by strati-graphy	mtDNA haplo-group
**XVII-B-125**	GbTo-23	M	Not Assigned	mand right 3rd molar	2260±40	cal BP 1870 to 1500	--	A*
**XVII-B-158**	GbTo-23	F	XX	mand left 3rd molar	2290±50	cal BP 2740 to 2350	--	A2
**XVII-B-163**	GbTo-18	M	XY	mand right 2nd molar	n/a	n/a	"prehistoric"	A2
**XVII-B-167**	GbTo-23	M	Not Assigned	mand right 2nd molar	n/a	n/a	cal BP 2750 to 2300	A*
**XVII-B-168**	GbTo-18	F	XX	molar	2650±75	cal BP 2330 to 1840	--	A2aq
**XVII-B-181**	GbTo-23	F	XX	mand left 3rd molar	2620±40	cal BP 2250 to 1770	--	A2d
**XVII-B-300**	GbTo-30	F	XX	mand left 3rd molar	1650±75	cal BP 1240 to 800	--	D1
**XVII-B-302**	GbTo-30	F	XX	mand left 3rd molar	2440±75	cal BP 2120 to 1600	--	A2
**XVII-B-311**	GbTo-31	M	Not Assigned	mand. right 3rd molar	2090±60	cal BP 1670 to 1280	--	A*
**XVII-B-318**	GbTo-31	M	Not Assigned	mand right 2nd molar	1550±50	cal BP 1170 to 800	--	D*
**XVII-B-322**	GbTo-31	M	Not Assigned	mand right 3rd molar	2050±50	cal BP 1620 to 1270	--	A*
**XVII-B-357**	GbTo-31	F	XX	mand right 3rd molar	n/a	n/a	cal BP 2000 to 1260	A2
**XVII-B-365**	GbTo-31	F	XX	molar	2270±65	cal BP 1890 to 1440	--	A*
**XVII-B-386**	GbTo-31	M	Not Assigned	mand left 2nd molar	1060±40	cal B 1036 to 946	--	A*
**XVII-B-406**	GbTo-31	F	XX	mand right 3rd molar	n/a	n/a	cal BP 2000 to 1260	A*
**XVII-B-412**	GbTo-31	F	Not Assigned	mand right 3rd molar	1940±40	cal BP 1490 to 1120	--	A2ao
**XVII-B-413**	GbTo-23	F	XX	molar	1970±42	cal BP 1500 to 1140	--	A2p
**XVII-B-443**	GbTo-31	M	Not Assigned	max left 2nd molar	1820±55	cal BP 1360 to 1000	--	A2p
**XVII-B-468**	GbTo-33	M	XY	mand right 3rd molar	1940±45	cal BP 1500 to 1130	--	A2p
**XVII-B-470**	GbTo-33	M	XY	mand right 3rd molar	1600±40	cal BP 1180 to 800	--	A2
**XVII-B-507**	GbTo-36	M	Not Assigned	mand right 3rd molar	2320± 65	cal BP 1890 to 1410	--	A*
**XVII-B-516**	GbTo-36	M	Not Assigned	mand right 2nd molar	n/a	n/a	cal BP 2250 to 1510	A2a
**XVII-B-525**	GbTo-31	M	Not Assigned	mand right 2nd molar	1860±40	cal BP 1340 to 990	--	A2p
**XVII-B-532**	GbTo-36	M	XY	molar	n/a	n/a	cal BP 3200 to 1500	A2p
**XVII-B-939**	GbTp-1	M	XX	mand left 2nd molar	5710±40	cal BP 6260 to 5890	--	D4h3a

Sequence-based sex determination was completed as described in Skoglund *et al.* [3]. The cal BP ranges were calculated as in [4] and account for the marine reservoir effect on the north coast of British Columbia. Mitochondrial haplogroups designated with * were assigned via restriction fragment length polymorphism. All others haplogroups are based on off target hits that aligned to the mitochondrial genome, which included the control region.

Supplementary Table 2 | Exome enrichment results

**Tsimshian Samples**	Exome Capture Method	Capture Libraries Sequenced	Illumina 62MB On-target Hits	Average Read Depth	**PRH Ancient Samples**	Exome Capture Method	Capture Libraries Sequenced	Illumina 62MB On-target Hits	Average Read Depth
S001	Nextera	1	0.904283758	6.3422	**125**	Tru-Seq	4	0.328200145	2.03254
S002	Tru-Seq	1	0.930950435	8.62802	**158**	Tru-Seq	4	0.536221871	5.25787
T004	Tru-Seq	1	0.705098484	2.80743	**163**	Tru-Seq	4	0.065611919	1.75727
T008	Nextera	1	0.801474597	10.6555	**167**	Tru-Seq	4	0.341898258	1.85701
T012	Tru-Seq	1	0.663371532	2.57248	**168**	Tru-Seq	4	0.84163529	12.8529
T015	Tru-Seq	1	0.90549279	9.87665	**181**	Tru-Seq	4	0.887003	12.7429
T018	Nextera	1	0.829727742	12.59	**300**	Tru-Seq	4	0.580635306	3.09703
T023	Tru-Seq	1	0.767662613	3.049	**302**	Tru-Seq	4	0.912515032	33.115
T024	Nextera	1	0.860356081	14.41	**311**	Tru-Seq	4	0.665996613	3.8207
T026	Nextera	1	0.917903548	7.61586	**318**	Tru-Seq	4	0.263561226	2.37845
T028	Nextera	1	0.862068403	4.83134	**322**	Tru-Seq	4	0.528905694	2.78005
T036	Tru-Seq	1	0.846779968	4.96615	**357**	Tru-Seq	4	0.772120629	10.0139
T052	Nextera	1	0.869650113	4.83776	**365**	Tru-Seq	4	0.851621435	13.2557
T054	Nextera	1	0.921754565	22.0136	**386**	Tru-Seq	4	0.326494726	1.66748
T055	Nextera	1	0.840876871	4.13476	**406**	Tru-Seq	4	0.477336452	2.71423
T057	Nextera	1	0.842303194	5.00326	**412**	Tru-Seq	4	0.326017484	1.65456
T058	Nextera	1	0.939344113	18.4778	**413**	Tru-Seq	4	0.716144032	6.33255
T059	Nextera	1	0.885633032	11.1151	**443**	Tru-Seq	4	0.864574258	27.2342
T060	Nextera	1	0.875366774	16.1518	**468**	Tru-Seq	4	0.914177242	17.9042
T061	Nextera	1	0.946905661	17.8558	**470**	Tru-Seq	4	0.929462935	15.2033
T066	Nextera	1	0.929130468	12.3193	**507**	Tru-Seq	4	0.602631839	2.78834
T067	Nextera	1	0.922596113	9.82765	**516**	Tru-Seq	4	0.710534081	6.07661
T200	Tru-Seq	1	0.818794145	4.52119	**525**	Tru-Seq	4	0.298353194	3.39217
T201	Tru-Seq	1	0.928923984	19.96	**532**	Tru-Seq	4	0.701980726	4.35001
T202	Tru-Seq	1	0.862945242	6.95669	**939**	Tru-Seq	4	0.342927726	4.93794

Supplementary Table 3 | PRH Ancient average read length and sample nuclear-based contamination estimates

PRH Ancient Sample	Average Read Length	Contamination Estimate Level at 95% Confidence	Confidence Interval 95 Low	Confidence Interval 95 High	Sites used for estimate
**125**	68	0.6%	0.4%	0.7%	3221
**158**	72	0.4%	0.3%	0.5%	3957
**163**	52	5.8%	5.4%	6.3%	2497
**167**	68	0.8%	0.7%	0.9%	3097
**168**	70	0.8%	0.8%	0.9%	2991
**181**	62	0.6%	0.6%	0.7%	2416
**300**	87	0.7%	0.7%	0.8%	3094
**302**	83	0.8%	0.6%	1.0%	3693
**311**	71	1.4%	1.1%	1.6%	2605
**318**	78	0.4%	0.4%	0.5%	3501
**322**	64	0.7%	0.7%	0.9%	2627
**357**	59	0.5%	0.5%	0.6%	3402
**365**	68	0.8%	0.8%	0.9%	3139
**386**	75	1.2%	0.8%	1.4%	3441
**406**	68	0.8%	0.8%	0.9%	3309
**412**	81	0.4%	0.4%	0.5%	2835
**413**	77	0.7%	0.7%	0.8%	3740
**443**	86	0.9%	0.6%	1.2%	3471
**468**	63	0.8%	0.8%	0.9%	2563
**470**	71	0.5%	0.4%	0.6%	3819
**507**	76	0.9%	0.7%	1.1%	2899
**516**	84	0.9%	0.7%	1.1%	2827
**525**	74	0.7%	0.5%	0.9%	3726
**532**	81	0.8%	0.7%	1.0%	2645
**939**	72	0.7%	0.6%	0.9%	2074

Contamination estimates were calculated with ContEst[5] and average read length was calculated withMapDamage2[6], using a minimum mapping quality score of 30.

Supplementary Table 4 | *HLA-DQA1* variant associated regulation information for SNPs showing the highest frequency change between the modern Tsimshian and the PRH Ancients.

chr	position (hg19)	function	variant ID	Ref	Alt	AFR Freq	AMR	ASN freq	EUR freq	
6	32605189	UTR5	rs9272426	A	G	0.28	0.52	0.49	0.39	
		**Tsimshian freq**	**Ancient PRH freq**	**Promoter histone marks**	**Enhancer histone marks**	**DNAse**	**Proteins bound**	**Motifs changed**	**NHGR/EBI GWAS hits**	**eQTL hits**
		0.37	1	blood, fat	blood	12 tissues	11 bound proteins	En-1	N/A	217 hits
**chr**	**position (hg19)**	**function**	**variant ID**	**Ref**	**Alt**	**AFR freq**	**AMR**	**ASN freq**	**EUR freq**	
6	32605197	UTR5	rs3207966	G	A	0.17	0.23	0.14	0.18	
		**Tsimshian freq**	**Ancient PRH freq**	**Promoter histone marks**	**Enhancer histone marks**	**DNAse**	**Proteins bound**	**Motifs changed**	**NHGR/EBI GWAS hits**	**eQTL hits**
		0.41	1	blood, fat	blood	10 tissues	11 bound proteins	GR,RFX5	N/A	107 hits
**chr**	**position (hg19)**	**function**	**variant ID**	**Ref**	**Alt**	**AFR freq**	**AMR freq**	**ASN freq**	**EUR freq**	
6	32605207	UTR5	rs3187964	C	G	0.42	0.66	0.55	0.55	
		**Tsimshian freq**	**Ancient PRH freq**	**Promoter histone marks**	**Enhancer histone marks**	**DNAse**	**Proteins bound**	**Motifs changed**	**NHGR/EBI GWAS hits**	**eQTL hits**
		0.55	0	7 tissues	blood, spleen	9 tissues	11 bound proteins	GLI, GR, Myf	N/A	293 hits
**chr**	**position (hg19)**	**function**	**variant ID**	**Ref**	**Alt**	**AFR freq**	**AMR freq**	**ASN freq**	**EUR freq**	
6	32605216	UTR5	rs1047985	G	A	0.2	0.25	0.14	0.2	
		**Tsimshian freq**	**Ancient PRH freq**	**Promoter histone marks**	**Enhancer histone marks**	**DNAse**	**Proteins bound**	**Motifs changed**	**NHGR/EBI GWAS hits**	**eQTL hits**
		0.43	1	N/A	N/A	9 tissues	11 bound proteins	GR,Myf,RFX5	N/A	109 hits
**chr**	**position (hg19)**	**function**	**variant ID**	**Ref**	**Alt**	**AFR freq**	**AMR freq**	**ASN freq**	**EUR freq**	
6	32605257	non-synonymous (missense)	rs1047989	C	A	0.37	0.59	0.5	0.52	
		**Tsimshian freq**	**Ancient PRH freq**	**Promoter histone marks**	**Enhancer histone marks**	**DNAse**	**Proteins bound**	**Motifs changed**	**NHGR/EBI GWAS hits**	**eQTL hits**
		0.55	1	N/A	N/A	5 tissues	9 bound proteins	5 altered motifs	N/A	266 hits
**chr**	**position (hg19)**	**function**	**variant ID**	**Ref**	**Alt**	**AFR Freq**	**AMR**	**ASN freq**	**EUR freq**	
6	32605271	synonymous	rs1047993	C	T	0.31	0.36	0.21	0.27	
		**Tsimshian freq**	**Ancient PRH freq**	**Promoter histone marks**	**Enhancer histone marks**	**DNAse**	**Proteins bound**	**Motifs changed**	**NHGR/EBI GWAS hits**	**eQTL hits**
		0.42	1	blood, fat	blood	12 tissues	11 bound proteins	N/A	N/A	161 hits

The table information was derived from HaploReg v4.1[7] and more detailed information on the eQTL hits for each variant (obtained from GTEx and 11 other databases) can be found at www.broad institute.org/mammals/haploreg. HaploReg reports frequencies and LD calculations (pairwise, within 250 kb) from the 1,000 Genomes Project, Phase 1. AMR=admixed North and South Americans, AFR=Africans, ASN=Asians, and EUR=Europeans.

Supplementary Table 5 | Functions of genes with *p*-values below 0.05 for the Tsimshian with correcting for European admixture

Gene	PBS score	*P*-value	#Sites	Function
PDCL3	0.299844400	0.02074	32	Phosducin-like 3. Acts as a chaperone for the angiogenic VEGF receptor KDR/VEGFR2, controlling its abundance and inhibiting its ubiquitination and degradation
CNOT11	0.173709579	0.04624	112	CCR4-NOT transcription complex, subunit 11. Component of the CCR4-NOT complex which is one of the major cellular mRNA deadenylases and is linked to various cellular processes including bulk mRNA degradation, miRNA-mediated repression.
PSG5	0.168520517	0.04818	16	Pregnancy specific beta-1-glycoprotein 5. The human pregnancy-specific glycoproteins (PSGs) are a group of molecules that are mainly produced by the placental syncytiotrophoblasts during pregnancy.
RAB6C	0.165826759	0.04936	12	Member RAS oncogene family.

Gene functions were derived from GeneCards [8]. *P*-values were calculated using neutral simulations. *See* Methods.

Supplementary Table 6 | Functions of Genes with *p*-values below 0.05 for the Tsimshian

Gene	PBS Score	*P*-value	#Sites	Function
CYP4Z1	0.329829344	0.01711	17	Mitochondrial translational release factor 1-like.
PDCL3	0.321767386	0.01797	39	Phosducin-like 3. Acts as a chaperone for the angiogenic VEGF receptor KDR/VEGFR2, controlling its abundance and inhibiting its ubiquitination and degradation. Modulates the activation of caspases during apoptosis.
PSG5	0.169863548	0.04913	19	Pregnancy specific beta-1-glycoprotein 5. The human pregnancy-specific glycoproteins (PSGs) are a group of molecules that are mainly produced by the placental syncytiotrophoblasts during pregnancy.
CNOT11	0.169410523	0.04938	160	CCR4-NOT transcription complex, subunit 11. Component of the CCR4-NOT complex which is one of the major cellular mRNA deadenylases and is linked to various cellular processes including bulk mRNA degradation, miRNA-mediated repression, translational repression during translational initiation and general transcription regulation.

Gene functions were derived from GeneCards[8]. *P*-values were calculated using neutral simulations. *See* Methods.

Supplementary Table 7 | Gene ontology enrichment terms for the PRH Ancients and modern Tsimshian from the PBS ranked list of genes

PRH Ancients PBS scan (corrected for admixture in the modern Tsimshian)		
Ontology enrichment term	*P* value	FDR *q* value
Olfactory receptor activity	3.17×10^{-9}	1.30×10^{-5}
MHC class II receptor activity	5.34×10^{-7}	1.09×10^{-3}
Peptide antigen binding	3.09×10^{-6}	4.21×10^{-3}
Receptor activity	1.26×10^{-5}	1.28×10^{-2}
G-protein coupled receptor activity	1.38×10^{-5}	1.13×10^{-2}
Peptidase inhibitor activity	4.36×10^{-5}	2.97×10^{-2}
Molecular transducer activity	5.13×10^{-5}	3.00×10^{-2}
Modern Tsimshian PBS scan		
No term reached significance (FDR *q*-value)		
Modern Tsimshian PBS scan (corrected for admixture)		
No term reached significance (FDR *q*-value)		

Data produced with GOrilla (http://cbl-gorilla.cs.technion.ac.il).

Supplementary Table 8 | Top 5 Genes in the PBS Scan involving the CHB, PEL, and PRH Ancients

Gene	PBS Score	#Sites	Function
RP11-16E12.2	0.360662415	7	Uncharacterized large intergenic non-coding RNA.
SMIM18	0.289866347	1	Small Integral Membrane Protein 18.
TMEM221	0.286607154	25	Transmembrane Protein 221
HLA-DQA1	0.258334539	34	MHC II Molecule
CELA3A	0.243607718	3	Protease secreted from the pancreas and has a digestive function in the intestine.

25 Peruvians were selected from phase 3 of the 1,000 Genomes Project, which showed little to no admixture. *HLA-DQA1* remains a top hit, indicating that European admixture in the modern Tsimshian is not skewing our results. Gene functions were derived from GeneCards[8]

**Supplementary Table 9 | Functions of genes with *p*-values below 0.05 for the PRH Ancients with admixture correction in the Tsimshian**				
Gene	**PBS**	***P*-value**	**# Sites**	**Function**
HLA-DQA1	0.278776809	0.02177	16	Major histocompatibility complex, class II, DQ alpha 1.
BLZF1	0.227968256	0.03116	56	Basic leucine zipper nuclear factor 1. Required for normal Golgi structure and for protein transport from the endoplasmic reticulum through the Golgi apparatus to the cell surface.
CELA3A	0.207270149	0.03651	27	Chymotrypsin-like elastase family, member 3A. Serine protease that hydrolyze many proteins in addition to elastin.
C1orf216	0.190562841	0.04162	65	Chromosome 1 open reading frame 216. Function not known.
OR6Q1	0.175425197	0.04809	12	Olfactory receptor, family 6, subfamily Q, member 1.
TOMM7	0.171970388	0.04982	19	Translocase Of Outer Mitochondrial Membrane 7 Homolog. Regulates the assembly and stability of the translocase complex.
Gene functions were derived from GeneCards[8].				

Supplementary Table 10 | Relationship between PRH Ancient individuals

125	158	163	167	168	181	300	302	311	318	322	357	365
125	NA	0.16942	0.0016472	0.0025068	0.0013219	-0.00149886	0.000361468	-0.00697741	-0.00419406	-0.00652168	0.0102084	0.0146706
158	0.16942	NA	-0.00120286	-0.00923655	-0.0281494	-0.0132274	-0.0118404	-0.0279142	-0.0193577	-0.0121542	0.0141206	0.0352277
163	0.0016472	-0.00120286	NA	0.00464826	0.00197754	0.00155458	0.00251313	-0.000527405	0.000425019	0.0335795	0.00265772	0.00379558
167	0.0025068	-0.00923655	0.00464826	NA	0.0148389	0.0928732	0.0139075	-8.66E-05	0.00304917	0.00123818	0.0177146	0.0233483
168	0.0013219	-0.0281494	0.00197754	0.0148389	NA	0.00639263	0.0299157	0.0363516	0.000424869	-0.00214166	0.0187918	0.0320527
181	-0.00149886	-0.0132274	0.00155458	0.0928732	0.00639263	NA	0.00486408	-0.0029634	-0.00116789	-0.00297599	0.0255548	0.0422006
300	0.000361468	-0.0118404	0.00251313	0.0139075	0.0299157	0.00486408	NA	-0.0053326	-0.000614363	-0.00321763	0.02501	0.0302731
302	-0.00697741	-0.0279142	-0.000527405	-8.66E-05	0.0363516	-0.0029634	-0.0053326	NA	-0.00975479	-0.00950484	0.00871614	0.0372684
311	-0.00419406	-0.0193577	0.000425019	0.00304917	0.000424869	-0.00116789	-0.000614363	-0.00975479	NA	-0.00730597	0.0201036	0.0304427
318	-0.00652168	-0.0121542	0.0335795	0.00123818	-0.00214166	-0.00297599	-0.00321763	-0.00950484	-0.00730597	NA	0.00927391	0.0125922
322	0.0102084	0.0141206	0.00265772	0.0177146	0.0187918	0.0255548	0.02501	0.00871614	0.0201036	0.00927391	NA	0.0610144
357	0.0146706	0.0352277	0.00379558	0.0233483	0.0320527	0.0422006	0.0302731	0.0372684	0.0304427	0.0125922	0.0610144	NA
365	0.00153287	-0.0213746	0.00115968	0.00949724	0.0257411	0.00867943	0.00536909	0.0190701	-0.000879507	-0.0034692	0.0200778	0.0597416
386	0.000127327	-0.0162206	0.00230807	0.00757377	0.0200497	-0.00110558	0.0097807	-0.00203702	9.11E-05	-0.000710934	0.0121723	0.0140133
406	0.000649197	-0.0101929	0.000171292	0.00564014	0.00365747	0.00362463	0.00643127	-0.00392864	0.000635377	-0.000353114	0.0207741	0.0284973
412	0.00186119	-0.0129701	0.00212529	0.00808096	0.00695327	-0.000356924	0.00226291	-0.0016714	0.000460397	-0.00269387	0.0135032	0.0155892
413	0.0139189	0.000901273	0.00308688	0.0196626	0.032196	0.022686	0.0277526	0.0265108	0.0212777	0.00993817	0.039347	0.0530426
443	0.00446414	-0.00373316	0.0020781	0.0084887	0.0293695	0.0177899	0.0101832	0.0438469	0.00832947	0.000998102	0.023562	0.0547492
468	-0.00124196	-0.0306631	0.000917872	0.000791248	0.0131108	-0.00174256	-0.0033413	0.0130178	-0.0090337	-0.00301654	0.0136407	0.0298022
470	-0.00171728	-0.0281616	-0.000276249	0.000925097	0.0134379	-0.00522956	-0.00332471	0.00943594	-0.00300824	-0.00705337	0.0172026	0.0288719
507	9.35E-05	-0.00827774	0.000610009	0.00506551	0.00477336	0.00389323	0.00553285	-0.00377886	0.00309548	0.000969007	0.0189601	0.0269137
516	-6.27E-06	-0.00609295	0.000679245	0.00414272	0.00226816	0.00195357	0.00536108	-0.00551506	0.00236436	0.000391036	0.0216858	0.0324815
525	-0.0308705	-0.0262513	-0.00477327	-0.0257816	-0.0532453	-0.0359601	-0.0387872	-0.0517576	0.0569931	-0.0287803	-0.00646682	-0.00152639
532	0.0048848	0.00701706	0.0019841	0.0102449	0.00848655	0.0124025	0.0143559	0.00507838	0.011861	0.0028907	0.162387	0.0502871
939	-0.129659	-0.122926	-0.037506	-0.135605	-0.326348	-0.238045	-0.208632	-0.310305	-0.22752	-0.148461	-0.0943614	-0.107981

125	386	406	412	413	443	468	470	507	516	525	532	939
125	0.00153287	0.000127327	0.000649197	0.00186119	0.0139189	0.00446414	-0.00124196	-0.00171728	9.35E-05	-6.27E-06	-0.0308705	0.0048848
158	-0.0213746	-0.0162206	-0.0101929	-0.0129701	0.000901273	-0.00373316	-0.0306631	-0.0281616	-0.00827774	-0.00609295	-0.0262513	0.00701706
163	0.00115968	0.00230807	0.000171292	0.00212529	0.00308688	0.0020781	0.000917872	-0.000276249	0.000610009	0.000679245	-0.00477327	0.0019841
167	0.00949724	0.00757377	0.00564014	0.00808096	0.0196626	0.0084887	0.000791248	0.000925097	0.00506551	0.00414272	-0.0257816	0.0102449
168	0.0257411	0.0200497	0.00365747	0.00695327	0.032196	0.0293695	0.0131108	0.0134379	0.00477336	0.00226816	-0.0532453	0.00848655
181	0.00867943	-0.00110558	0.00362463	-0.000356924	0.022686	0.0177899	-0.00174256	-0.00522956	0.00389323	0.00195357	-0.0359601	0.0124025
300	0.00536909	0.0097807	0.00643127	0.00226291	0.0277526	0.0101832	-0.0033413	-0.00332471	0.00553285	0.00536108	-0.0387872	0.0143559
302	0.0190701	-0.00203702	-0.00392864	-0.0016714	0.0265108	0.0438469	0.0130178	0.00943594	-0.00377886	-0.00551506	-0.0517576	0.00507838
311	-0.000879507	9.11E-05	0.000635377	0.000460397	0.0212777	0.00832947	-0.0090337	-0.00300824	0.00309548	0.00236436	0.0569931	0.011861
318	-0.0034692	-0.000710934	-0.000353114	-0.00269387	0.00993817	0.000998102	-0.00301654	-0.00705337	0.000969007	0.000391036	-0.0287803	0.0028907
322	0.0200778	0.0121723	0.0207741	0.0135032	0.039347	0.023562	0.0136407	0.0172026	0.0189601	0.0216858	-0.00646682	0.162387
357	0.0597416	0.0140133	0.0284973	0.0155892	0.0530426	0.0547492	0.0298022	0.0288719	0.0269137	0.0324815	-0.00152639	0.0502871
365	NA	0.00193385	0.00253133	0.00232335	0.0353296	0.0342017	0.0117768	0.0116719	0.00555632	0.0043735	-0.0454185	0.0104558
386	0.00193385	NA	0.00148496	0.0075188	0.0194528	0.00728132	0.000480151	0.0984132	0.00251937	0.00180859	-0.0347256	0.00585642
406	0.00253133	0.00148496	NA	0.000887778	0.0214383	0.00788187	-0.00354749	-0.00295042	0.0574437	0.0958468	-0.0264523	0.0124907
412	0.00232335	0.0075188	0.000887778	NA	0.0189004	0.00766337	0.135905	-0.00165084	0.00242144	0.00143596	-0.030953	0.00670642
413	0.0353296	0.0194528	0.0214383	0.0189004	NA	0.180184	0.0287762	0.0262798	0.0191697	0.018399	-0.0221565	0.0289197
443	0.0342017	0.00728132	0.00788187	0.00766337	0.180184	NA	0.0273045	0.0261457	0.0079353	0.00964367	-0.0295058	0.019091
468	0.0117768	0.000480151	-0.00354749	0.135905	0.0287762	0.0273045	NA	0.0040607	-0.000859949	-0.00453764	-0.0550928	0.00399181
470	0.0116719	0.0984132	-0.00295042	-0.00165084	0.0262798	0.0261457	0.0040607	NA	-0.00128567	-0.00449368	-0.0515996	0.00598947
507	0.00555632	0.00251937	0.0574437	0.00242144	0.0191697	0.0079353	-0.000859949	-0.00128567	NA	0.111719	-0.0149821	0.0120985
516	0.0043735	0.00180859	0.0958468	0.00143596	0.018399	0.00964367	-0.00453764	-0.00449368	0.211719	NA	-0.0142088	0.0126648
525	-0.0454185	-0.0347256	-0.0264523	-0.030953	-0.0221565	-0.0295058	-0.0550928	-0.0515996	-0.0149821	-0.0142088	NA	0.00192353
532	0.0104558	0.00585642	0.0124907	0.00670642	0.0289197	0.019091	0.00399181	0.00598947	0.0120985	0.0126648	0.00192353	NA
939	-0.288325	-0.174031	-0.143808	-0.163988	-0.191826	-0.200822	-0.327921	-0.331492	-0.0854724	-0.0910947	-0.133344	-0.138544

Relatedness was assessed using KING [9], where the kinship coefficient Φ for ranges of >0.354, 0.177-0.354, 0.0884-0.177, 0.0442-0.0884 correspond to duplicate/MZ twin, first-degree, second-degree, and third-degree relationships, respectively. Pairwise comparisons revealed no inferred close relatives, except for PRH 516 and PRH 507, where a possible second-degree relationship was inferred. However, it should be noted that these two individuals are temporally separated by hundreds of years, and therefore this inferred relationship is likely a false positive.

Supplementary Note 1 –Archeological site descriptions

Archaeology of Prince Rupert Sites, British Columbia

Twenty-four of the 25 ancient individuals tested in this study are from six inner harbour sites near the city of Prince Rupert where major archaeological excavations were carried out by the National Museum of Canada (now the Canadian Museum of History) between 1966 and 1973[10]. They include site numbers GbTo-18, -23, -30, -31, -33, and -36 as shown on Supplementary Figure 1. The 25th individual came from GbTp-1 located in the Lucy Islands about 17 km west of the inner harbor sites (Fig. 1 in Cui et al. [11]; details in [12]).

All seven archaeological sites are ancient shell middens that represent hundreds and thousands of years of culture history. In addition to their evidence for habitation, subsistence (food remains and food getting technology), woodworking tools, and the arts, the middens have traditionally served as cemeteries apparently with ritual significance[13]. At the time of European contact, ten tribes of the Skeena River or Coast Tsimshian laid claim to various localities in and about the inner harbor and the Lucy Islands (Halpin and Seguin 1990). Their way of life is reflected by the contents of the middens, a finding which suggests long-term ancestral occupation. Radiocarbon dates indicate that the Prince Rupert Harbour region was continuously occupied from about cal BP 6000 to 350 BP[4] with a potentially earlier occupation inferred[14]. Specific radiocarbon dates for most of the tested human remains are reported in Supplementary Table 1.

Supplementary Note 2 – Ancestry analyses

Multi-dimensional scaling (MDS) applied to called genotypes

We intersected the called genotypes for the 25 ancient and 25 modern exomes from this study and from the Mayan, Surui, and Karitiana exomes from Szpiech et al. [28] with called genotypes from whole genome sequences from the 1,000 Genome Project Phase 2 samples[23], the Saqqaq ancient sample from Rasmussen et al. [29], and the Anzick-1 ancient sample from Rasmussen et al. [1]. To guard against biases generated by post-mortem deamination, sites where a C/T or G/A polymorphism was observed were removed. Further, sites that were not biallelic were also removed. In addition, only sites for which each population was not completely missing data were retained. A total of 29,333 polymorphic loci were employed for this analysis.

For each site k in the filtered dataset, we calculated a distance d_{ij}^{k} between individuals i and j, where $d_{ij}^{k} = 1$ if the pair of individuals had different homozygous genotypes, $d_{ij}^{k} = 0.5$ if one of the pair was homozygous and the other was heterozygous, and $d_{ij}^{k} = 0$ if both individuals were homozygous for the same genotype. If at least one of the individuals has missing data at site k, then $d_{ij}^{k} = 0$. Let L be the number of overlapping sites in the filtered dataset, and suppose L_{ij} is the number of sites at which individuals i and j both have non-missing data. We define the allele sharing distance between individuals i and j as

$$d_{ij} = \frac{1}{L_{ij}} \sum_{k=1}^{L} d_{ij}^{k}.$$

We then applied classical multi-dimensional scaling to the matrix defined by this set of pairwise distances.

Plotting the first two components reveals that the first component separates out Africans from East Asians, and the second component separates out Africans and East Asian from Europeans. The modern Central and South American populations (Surui, Karitiana, and Mayan) fall closest to the East Asians, with the ancient Anzick-1 sample from Montana and the PRH Ancient individuals from this study falling near the modern Central and South Americans. In contrast, though the modern Tsimshian fall closest to the modern Central and South American and the other ancient samples from the Americas, they also lie intermediate between the modern Native American and modern European samples.

Assessment of population structure using *ADMIXTURE*
We started with the identical filtered dataset of called genotypes described in the Methods. We further pruned the dataset by removing sites in strong linkage disequilibrium ($r^2 > 0.1$) using PLINK[2]. The program *ADMIXTURE*[30] was used to assess global ancestry of the ancient and present-day samples from this study. We computed cluster membership for K=2, through K=5 clusters, as displayed in Figure 1a. A total of 29,333 polymorphic loci were employed for this analysis.

At K=5, the ancient samples separate into their own cluster, depicted in gray (Fig. 1a). This cluster is also the major ancestry component of the present-day samples from this study, and is also a large proportion of other ancient and modern Native Americans (i.e., Anzick-1, Maya, and Surui). It should be noted that this genetic component (gray) decreases as the population sampling locations move south, whereas the ancient Saqqaq sample from Greenland share little, potentially suggesting a different migration wave. Further, the modern Tsimshian samples from this study display a large ancestry component matching Europeans, which is consistent with admixture from European contact with the Americas. This result is also reflected in the MDS analysis (Fig. 1b), in which the modern Tsimshian individuals have ancestry intermediate between other Native American groups and Europeans.

Supplementary Note 3 – SNP validation and gene functions

HLA-DQA1 SNP validation and variant location

The *HLA-DQA1* SNP showing the highest frequency changes (located at positions chr6: 32605189 and chr6: 32605197) between the PRH Ancients and Tsimshian were confirmed via Sanger sequencing from 18 of the ancient individuals (Supplementary Fig. 4). The remaining 7 ancient samples either did not amplify in the specified region or the sequence was not readable. The extraction method was the same as described above. Forward and reverse PCR primers were constructed as follows: CCTCACAATTACTCTACAGCTCAG and CTCATGCACTCACCCACAA.

PCR reactions were performed with 25ul of Q5 High-Fidelity 2X master mix (New England Biolabs, Ipswich, MA), 0.2µM of each PCR primer, 3% DMSO (New England Biolabs, Ipswich, MA), and 0.2mg/ml BSA (New England Biolabs, Ipswich, MA). PCR conditions were as follows: 30s at 98°C, 50 cycles of 10s at 98°C, 30s at 60°C and 30s at 72°C, with a final extension at 72°C for 2m.

Gene function of genes putatively under positive selection
Gene function descriptions derived from GeneCards[8]. *See* Supplementary Tables 5, 6, 8, and 9.

Gene ontology enrichment

Gene ontologies were performed using the ranked list of genes from the PBS scan and produced with GOrilla (http://cbl-gorilla.cs.technion.ac.il) [31]. *See* Supplementary Table 7.

Supplementary Note 4 – Assessment of biases from capture probes

We examined the possible effects of reference bias in our data due to the capture probe design. We chose the two samples with average coverage greater than 20x, samples 302 and 443, and called heterozygous sites with Samtools. Only sites with 20 reads or higher were kept. The distribution of the proportion of reads matching the reference, across SNPs in the exome, were plotted in Supplementary Figure 23. The two ancient samples show a slight bias, both showing a mean proportion of ~0.56 in favor of the reference allele. This bias could be attributed to differential mapping and potentially the design of the capture probes. However, this bias does not correlate with our top PBS hit, since the SNPs are all called for the *alternative* allele to the reference.

Supplementary Note 5 – DNA extraction and library prep

DNA extraction

DNA extractions and PCR amplification setups were completed in an ancient DNA laboratory facility at the University of Illinois Urbana-Champaign. The ancient DNA lab is a positively pressured clean room with hepa-filtered air. The clean room contains an anteroom and air flows from the ancient DNA lab to the anteroom to the hallway. Personnel working in the ancient DNA lab wear disposable hairnets, facemasks, laboratory coveralls and booties. All equipment, reagents and consumables are dedicated for use in the ancient DNA laboratory. The ancient DNA lab is routinely cleaned with bleach and all containers are wiped with Takara DNA Off (Mountain View, CA) before placed in the ancient DNA lab. Personnel are restricted from entering the ancient DNA after being in a modern DNA laboratory. A database containing mitochondrial control region sequence is maintained of all personnel working in the ancient laboratory and of any personnel who may have come into contact with the human remains prior to DNA analysis. Contamination controls were used with every DNA extraction and PCR setup in order to detect any contamination from reagents. A series of negative controls are routinely performed in the ancient DNA lab.

Each tooth was soaked in 6% sodium hypochlorite for 3 minutes, rinsed three times with UV-irradiated molecular grade water, and dried in a UV Crosslinker for 20 minutes, so as to remove surface contamination. Approximately 0.20 grams of tooth powder was incubated in 4 ml of demineralization/lysis buffer (0.5 M EDTA, 33.3 mg/ml Proteinase K, 10% N-lauryl sarcosine) for 24 hours at 37ºC. The digested sample was then concentrated to approximately 250 **μ**l using Amicon centrifugal filter units. Following concentration, the digest was run through silica columns using the MinElute Qiagen PCR Purification Kit (Qiagen, Hilden, Germany) and eluted in 60 **μ**l of DNA extract.

DNA screening for mtDNA

In order to test for viable DNA before proceeding with library building and exome sequencing, each ancient individual was amplified for the hypervariable region I of mitochondrial DNA from 2 **μ**l of extract, utilizing the reagents and conditions described in Malhi *et al.* [15]. Native American

maternal haplogroup was also confirmed for each sample via restriction fragment length polymorphism analysis for Native American haplogroups (A, B, C, D, and X)[16], or via off-target reads from the exome capture and previously performed captures of the mitochondrial genome, as described in Cui *et al.* [11] (Supplementary Table 1). All 25 ancient samples demonstrated Native American mitochondrial haplogroups.

Library build

Libraries were created in the ancient lab facility using the New England Biolabs Ultra Kit for Illumina (E7370S, Ipswich, MA) following the manufacturer's protocol with the following modifications. DNA fragmentation was not performed. DNA purifications were done using the MinElute Reaction Cleanup Kit (Qiagen, Valencia, CA). Library amplification was done in two steps. The first round of amplification utilized the kit's reagents and protocol with 12 cycles of (10s at 98°C, 30s at 65°C and 30s at 72°C). For the second round, we achieved a sufficient DNA concentration for the exome enrichment (~500ng), without excessive amplification, by creating 4 PCR reactions from the initial amplified product and then pooling them before using a Qiagen MinElute PCR Clean-up kit. For the 2nd PCR, we created a 50 **µ**l reaction, utilizing 0.2µM of primers P5 (5'- AATGATACGGCGACCACCGA) and P7 (5' CAAGCAGAAGACGGCATACGA) [17], 5µl from the initial PCR, 25**µ**l of Phusion® High-Fidelity PCR Master Mix with HF Buffer (New England Biolabs, Ipswich, MA), 3% DMSO (New England Biolabs, Ipswich, MA), 0.2mg/ml BSA (New England Biolabs, Ipswich, MA). PCR conditions were as follows: 4m at 98°C, 10 cycles of (10s at 98°C, 30s at 62°C and 30s at 72°C), with a final extension at 72°C for 10m. Library fragment sizes were confirmed via a BioAnalyzer High Sensitivity assay to be above 130bp.

Copenhagen sample sequencing

PRH Ancient samples 406 and 507 were extracted, libraries built, and sequenced at the Centre for GeoGenetics in Copenhagen, Denmark, using the protocols described in Rasmussen et al. [18]. The exome enrichment was also conducted at the Copenhagen facility utilizing the Illumina TruSeq Exome Enrichment Kit (Illumina, San Diego, CA), with the protocol modifications described above.

Supplementary Note 6 – Mapping and damage patterns

Mapping

Raw data from the Illumina HiSeq 2000 platform was base called with CASAVA 1.8.2. Sequences were de-multiplexed with a requirement for a full match of the six nucleotide indexes that was used for library preparation. Illumina adapter sequences were trimmed using Trimmomatic-0.32[19] with a minimum length of 25 and removing leading and trailing quality or N bases below a quality score of 3. Reads were additional trimmed for 5 bp at each end to minimize transitions due to DNA damage. Trimmed reads were aligned to the human reference genome, Hg19, HS Build37.1, using bwa[20] with seeding disabled and with parameters set according to published recommendations for ancient DNA[21]. SAMtools-1.1[22] was used to sort and remove duplicate reads based on mapping positions.

Our bioinformatics pipeline was confirmed for accuracy by sequencing and processing two samples from the 1,000 Genomes Project [23] with the methods described above. DNA samples from NA18524 and NA18486 were retrieved from the NHGRI sample repository (Coriell Institute, Camden, NJ). The results were plotted on an MDS plot with the HGDP-CEPH

Diversity Panel[24] mapped to Hg19, and the two samples clustered with their expected populations (Fig S2).

DNA damage patterns

DNA damage (type I and type II) was assessed by comparing T→C/G→A and C→T/A →G transitions, respectively using MapDamage 2.0[6]. A specific pattern of DNA damage has been identified in other ancient DNA studies[25,26]. These studies show a pattern of increased type II DNA damage at the beginning and end of degraded DNA fragments. The MapDamage results show signatures of DNA damage, consistent with use of both the AT overhang library technique and blunt end[27], which suggests that the ancient sequences originate from ancient DNA templates and not modern contaminants (Supplementary Fig. 2).

Supplementary Note 7 – Relatedness analysis

Relatedness of the Ancient PRH individuals was assessed using KING[9], where the kinship coefficient Φ for ranges of >0.354, 0.177-0.354, 0.0884-0.177, 0.0442-0.0884 correspond to duplicate/MZ twin, first-degree, second-degree, and third-degree relationships, respectively. Pairwise comparisons revealed no inferred close relatives, except for PRH 516 and PRH 507, where a possible second-degree relationship was inferred (Supplementary Table 10). However, it should be noted that these two individuals are temporally separated by hundreds of years, and therefore this inferred relationship is likely a false positive.

Supplementary Note 8 – PBS Selection Scans

We performed two scans for positive selection. The first was a per-gene scan, in which we calculated PBS for the PRH Ancient population (PBS_{PRH}) using all data at given gene. Gene annotations were derived from RefSeq, utilizing the longest transcript for a given gene. The transcript length was taken as the transcription start to the transcription stop, and included both introns and exons. F_{ST} between each pair of populations was calculated using all SNPs that fell between the transcription start and stop of the gene, as well as 10 kilobases upstream of the transcription start and 10 kilobases downstream of the transcription stop (similar to how it was performed in Huerta-Sánchez et al. [32]). We then ranked each gene in the genome with decreasing PBS. The top two candidates are displayed in Table 2.

The second scan was a per-SNP scan, in which we calculated PBS for the PRH Ancient population (PBS_{PRH}) and the modern Tsimshian population ($PBS_{Tsimshian}$) at each SNP. That is, F_{ST} between each pair of populations was calculated for a given SNP, and this set of F_{ST} values was used to calculate PBS for that SNP. We then created Manhattan plots, and highlight chromosome 6 in Figure 3.

We also performed an analogous scan in which we substituted 25 mostly unadmixed Peruvian samples from the 1,000 Genomes Project Phase 3[1] for the 25 modern Tsimshian individuals. We identified the individuals showing little to no admixture by running *ADMIXTURE*[30]. The individuals identified and used for this and subsequent analyses were: HG01572, HG01923, HG01926, HG01927, HG01941, HG01951 ,HG01953, HG01954, HG02008, HG02102, HG02105, HG02146, HG02147, HG02150, HG02259, HG02260, HG02266, HG02271, HG02272, HG02275, HG02278, HG02291, HG02292, HG02304, HG02348. Because the individuals appeared mostly unadmixed, we did not correct allele frequencies for admixture.

Results from this scan are highlighted in Supplementary Table 8. Using his different reference sister Native American population still suggests that *HLA-DQA1* is a reasonable candidate, as it is ranked fourth in the scan—with the three genes ranked above it devoid of functional characterization—using Peruvians rather than modern Tsimshian as a reference sister population.

Supplementary References

1. Altshuler, D. M. *et al.* A global reference for human genetic variation. *Nature* **526,** 68–74 (2015).
2. Maples, B. K., Gravel, S., Kenny, E. E. & Bustamante, C. D. RFMix: A Discriminative Modeling Approach for Rapid and Robust Local-Ancestry Inference. *The American Journal of Human Genetics* **93,** 278–288 (2013).
3. Skoglund, P., Stora, J., Gotherstrom, A. & Jakobsson, M. Accurate sex identification of ancient human remains using DNA shotgun sequencing. *Journal of Archaeological Science* **40,** 4477–4482 (2013).
4. Cybulski, J. S. in *Violence and Warfare Among Hunter-Gatherers* (eds. Allen, M. W. & Jones, T. L.) 333–350 (2014).
5. Cibulskis, K. *et al.* ContEst: estimating cross-contamination of human samples in next-generation sequencing data. *Bioinformatics* **27,** 2601–2602 (2011).
6. Jonsson, H., Ginolhac, A., Schubert, M., Johnson, P. L. F. & Orlando, L. mapDamage2.0: fast approximate Bayesian estimates of ancient DNA damage parameters. *Bioinformatics* **29,** 1682–1684 (2013).
7. Ward, L. D. & Kellis, M. HaploReg: a resource for exploring chromatin states, conservation, and regulatory motif alterations within sets of genetically linked variants. *Nucleic Acids Research* **40,** D930–4 (2012).
8. Safran, M. *et al.* GeneCards Version 3: the human gene integrator. *Database (Oxford)* **2010,** baq020(2010).
9. Manichaikul, A. *et al.* Robust relationship inference in genome-wide association studies. *Bioinformatics* **26,** 2867–2873 (2010).
10. MacDonald, G. F. & Cybulski, J. S. in *Perspectives on Northern Northwest Coast Prehistory* (ed. Cybulski, J. S.) 1–23 (Gatineau, Quebec : Canadian Museum of Civilization, 2001).
11. Cui, Y. *et al.* Ancient DNA Analysis of Mid-Holocene Individuals from the Northwest Coast of North America Reveals Different Evolutionary Paths for Mitogenomes. *PLoS One* **8,** e66948 (2013).
12. Cybulski, J. S. Human Remains from Lucy Island, British Columbia, Site GbTp 1, 1984/85. *Prepared for Museum of Northern British Columbia, Canadian Museum of History Archives Ms.* **2360,** (1986).
13. Cybulski, J. S. A Greenville Burial Ground; Human Remains and Mortuary Elements in British Columbia Coast Prehistory. *Mercury Series Archaeology Paper* **146,** (1992).
14. Ames, K. *The North Coast Prehistory Project Excavations in Prince Rupert Harbour, British Columbia: The Artifacts*. *British Archaeological Reports BAR International Series* **1342,** (2005).
15. Malhi, R. S. *et al.* Native American mtDNA prehistory in the American Southwest. *Am. J. Phys. Anthropol.* **120,** 108–124 (2003).
16. Kaestle, F. A. & Smith, D. G. Ancient mitochondrial DNA evidence for prehistoric population movement: the Numic expansion. *Am. J. Phys. Anthropol.* **115,** 1–12 (2001).
17. Meyer, M. & Kircher, M. Illumina Sequencing Library Preparation for Highly Multiplexed Target Capture and Sequencing. *Cold Spring Harbor Protocols* **2010,** pdb.prot5448–pdb.prot5448 (2010).
18. Rasmussen, M. *et al.* The genome of a Late Pleistocene human from a Clovis burial site in western Montana. *Nature* **506,** 225–229 (2014).
19. Bolger, A. M., Lohse, M. & Usadel, B. Trimmomatic: a flexible trimmer for Illumina sequence data. *Bioinformatics* **30,** 2114–2120 (2014).
20. Li, H. & Durbin, R. Fast and accurate short read alignment with Burrows–Wheeler transform. *Bioinformatics* **25,** 1754–1760 (2009).
21. Schubert, M. *et al.* Improving ancient DNA read mapping against modern reference genomes. *BMC Genomics* **13,** 178 (2012).
22. Li, H. *et al.* The Sequence Alignment/Map format and SAMtools. *Bioinformatics* **25,** 2078–2079 (2009).
23. 1000 Genomes Project Consortium *et al.* An integrated map of genetic variation from 1,092 human genomes. *Nature* **491,** 56–65 (2012).
24. Cann, H. M. *et al.* A human genome diversity cell line panel. *Science* **296,** 261–262 (2002).
25. Rasmussen, M. *et al.* An Aboriginal Australian Genome Reveals Separate Human Dispersals into Asia. *Science* **334,** 94–98 (2011).
26. Rasmussen, M. *et al.* Ancient human genome sequence of an extinct Palaeo-Eskimo. *Nature* **463,** 757–762 (2010).
27. Seguin-Orlando, A. *et al.* Ligation Bias in Illumina Next-Generation DNA Libraries: Implications for Sequencing Ancient Genomes. *PLoS One* **8,** e78575 (2013).
28. Szpiech, Z. A. *et al.* Long Runs of Homozygosity Are Enriched for Deleterious Variation. *The American Journal of Human Genetics* **93,** 90–102 (2013).

29. Browning, S. R. & Browning, B. L. Rapid and Accurate Haplotype Phasing and Missing-Data Inference for Whole-Genome Association Studies By Use of Localized Haplotype Clustering. *The American Journal of Human Genetics* **81,** 1084–1097 (2007).
30. Alexander, D. H., Novembre, J. & Lange, K. Fast model-based estimation of ancestry in unrelated individuals. *Genome Research* **19,** 1655–1664 (2009).
31. Eden, E., Navon, R., Steinfeld, I., Lipson, D. & Yakhini, Z. GOrilla: a tool for discovery and visualization of enriched GO terms in ranked gene lists. *BMC Bioinformatics* **10,** 48 (2009).
32. Huerta-Sanchez, E. *et al.* Genetic Signatures Reveal High-Altitude Adaptation in a Set of Ethiopian Populations. *Molecular Biology and Evolution* **30,** 1877–1888 (2013).
